# Valve-sparing Aortic Root Replacement: Defining High-volume Centres Using Prospective Data

**DOI:** 10.1093/ejcts/ezag177

**Published:** 2026-05-22

**Authors:** Pascal J Govers, Michal J Kawczynski, Samuel Heuts, Kevin M Veen, Laurent de Kerchove, Emmanuel Lansac, Philippe Demers, Peter Verbrugghe, Igor Rudez, Aline Laubriet, Jan Vojacek, Elham Bidar, Johanna Takkenberg, Jolanda Kluin, Bardia Arabkhani, the Aortic Valve Research Network Investigators, Claudia Romagnoni, Frederiek de Heer, Jesper Hjortnaes, Adrián Kolesár, Alejandro Crespo de Hubsch, Christian Dinges, Jaroslav Hlubocky, Carlotta Brega, Ruggero de Paulis, Mauro Masat, Francesco Patane, Matteo Pettinari, Maciej Matuszewski, Gianclaudio Mecozzi, Jan Nijs, Marek Jasinski, Javier Estigarribia, Massimo Mariani, Pallav Shah, Diana Aicher, Didier Chatel, Aayush Poddar, Vladislav Aminov, Eric Bergoend, Aude Boignard, Guillaume Geuzebroek, Takeshi Miyairi, German Chaud, Ignacio Bibiloni, Evaldas Girdauskas, Guido van Aarnhem, Said Soliman, Mladen Kocica, Alberto Forteza, Carlos Porras, Plamen Panayotov, Yutaka Okita, Thierry Bourguignon, Corinne Coulon, Nadia Mansour, Kathy Louro, Vincent Chauvette, Bart Meuris, Davor Baric, Daniel Unic, Olivier Bouchot, Mikita Karalko, Pavel Žáček, Rubina Rosa, Andrea Mangini, Tomáš Toporcer, Johannes Steindl, Robert Novotny, Andrey Slautin, Giulio Folino, Fabrizio Ceresa, Carlijn van der Ven, Patrick Yiu, Ryan Accord, Cristobal Alvarado, Joaquin Gundelach, Gregorio Rábago, Leona Schultz, Dejan Lazovic, Carlos Martin, Gemma Sánchez-Espín, Milen Slavov

**Affiliations:** Department of Cardiothoracic Surgery, Erasmus University Medical Center, Rotterdam, 3015GD, The Netherlands; Department of Cardiothoracic Surgery, Maastricht University Medical Center (MUMC+), Maastricht, 6229HX, The Netherlands; Cardiovascular Research Institute Maastricht (CARIM), Maastricht University, Maastricht, 6229ER, The Netherlands; Department of Cardiothoracic Surgery, Maastricht University Medical Center (MUMC+), Maastricht, 6229HX, The Netherlands; Cardiovascular Research Institute Maastricht (CARIM), Maastricht University, Maastricht, 6229ER, The Netherlands; Department of Cardiothoracic Surgery, Erasmus University Medical Center, Rotterdam, 3015GD, The Netherlands; Division of Cardiothoracic and Vascular Surgery, Cliniques Universitaires Saint-Luc, Université Catholique de Louvain, Brussels, 1200, Belgium; Department of Cardiac Surgery, Institute Mutualiste Montsouris, Paris, 75014, France; Department of Cardiac Surgery, Montreal Heart Institute, Université de Montréal, Montréal, Québec, H1T 1C8, Canada; Cardiac Surgery, KU Leuven University Hospitals Leuven, Leuven, 3000, Belgium; Department of Cardiac and Transplant Surgery, University Hospital Dubrava, Zagreb, 10000, Croatia; Department of Cardiovascular and Thoracic Surgery, Dijon University Hospital, Dijon, 21000, France; Department of Cardiac Surgery, Charles University, Faculty of Medicine and University Hospital in Hradec Kralove, Hradec Kralove, 500 05, Czech Republic; Department of Cardiothoracic Surgery, Maastricht University Medical Center (MUMC+), Maastricht, 6229HX, The Netherlands; Cardiovascular Research Institute Maastricht (CARIM), Maastricht University, Maastricht, 6229ER, The Netherlands; Department of Cardiothoracic Surgery, Erasmus University Medical Center, Rotterdam, 3015GD, The Netherlands; Department of Cardiothoracic Surgery, Erasmus University Medical Center, Rotterdam, 3015GD, The Netherlands; Department of Cardiothoracic Surgery, Erasmus University Medical Center, Rotterdam, 3015GD, The Netherlands

**Keywords:** valve-sparing operations, volume-outcome association, annual case volume, optimal case volume, reimplantation technique, aortic root surgery

## Abstract

**Objectives:**

Valve-sparing aortic root replacement (VSARR) is technically demanding and may vary in institutional procedural volumes, rendering the establishment of thresholds for centres of expertise challenging. This study aims to identify an annual case volume that could define a high-volume centre for VSARR using data from the Heart Valve Society Aortic Valve (HVS AV) database.

**Methods:**

All consecutive elective patients undergoing VSARR within the HVS AV database were included. The primary early outcome was a composite of mortality, thromboembolic events, reinterventions, intraoperative conversions, and recurrent aortic regurgitation grade ≥2 within 30 days. The primary long-term outcome was freedom from AV reintervention. Volume-outcome (V-O) associations were modelled using restricted cubic splines; the high-volume threshold was derived using the elbow method. Analyses were adjusted for EuroSCORE II.

**Results:**

In total, 2668 patients from 37 centres were included. Annual volume was not significantly associated with the early composite outcome (*P* = .8003). However, a significant non-linear V-O association was found for long-term AV reintervention-free survival (*P* = .0023), with improved outcomes at centres performing ≥12 cases per year (95% CI, 10-12). These results were consistent in sensitivity analyses (*P* < .0001).

**Conclusions:**

An annual institutional volume of ≥12 VSARRs is associated with improved long-term valve durability and survival, while early postoperative outcomes appear less sensitive to annual case load given their low event rates. This high-volume threshold may serve as a benchmark for centres already experienced in AV and/or root surgery and guide quality improvement efforts.

## Graphical abstract

**Figure:**
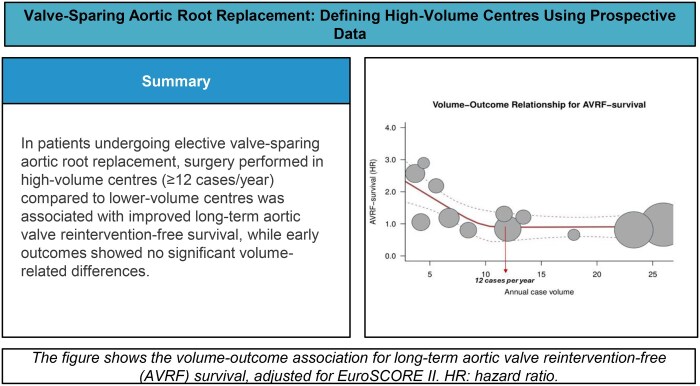

## Introduction

Valve-sparing aortic root replacement (VSARR) preserves the patient’s native aortic valve (AV), eliminating the need for lifelong anticoagulation and maintaining natural haemodynamics. Despite its proven clinical benefits in appropriately selected patients, VSARR is a technically demanding procedure that is performed relatively infrequently and carries a long-term risk of reintervention.^1–3^ Currently, VSARR is performed in centres of varying annual volumes, and recommendations on annual caseloads required to perform VSARR are lacking.

Centralization of specialized healthcare services has become a pivotal aspect to improving the quality of medical procedures. Conceptually, by concentrating cases in higher-volume centres, surgeons and medical teams become more experienced, which has been associated with improved patient outcomes across various medical disciplines.^4^ This association between volume-outcome (V-O) has been well-documented, leading to the establishment of specific annual case volume thresholds that define centres of excellence for several procedures.^5–9^ However, determining these thresholds for complex procedures like VSARR poses significant challenges due to limited procedural frequency, low event rates, and data variability.

We evaluated V-O associations for VSARR using prospective registry data from the Heart Valve Society Aortic Valve (HVS AV) Database and aimed to identify a data-driven annual case volume threshold to define high-volume centres.

## Methods

### Ethical statement

Access to the HVS AV Database was granted by the HVS Scientific Committee. Participating centres obtained approval from their local ethics committee/IRB or a formal waiver. Written consent was obtained when required and waived where permitted. Erasmus MC approved participation (MEC-2020-0890). Data handling followed each site’s regulations and HVS AV Database governance. The HVS AV Database, and consequently this study, follows the WMA Declarations of Helsinki (2013) and Taipei (2016).

### Protocol and registration

This study is an analysis of prospectively collected registry data from the HVS AV Database. This registry, including governance, has been described in detail elsewhere.^10^ The protocol was reviewed by the HVS AV Database Scientific Committee prior to data release. De-identified data were extracted on October 14, 2024 for all participating centres contributing VSARR cases within the inclusion window.

### Data collection

All elective primary VSARR cases with complete data on timing of death, reintervention, and early postoperative outcomes were included in the study. Patients in the HVS AV database were operated on between 1996 and 2023. A CONSORT-style flow diagram illustrating the selection of patients from the HVS AV database is provided in **Supplementary Material S1**.

### Outcome measures

The primary outcomes were the association between annual institutional volume and (i) early postoperative outcomes (in-hospital or within 30 days after surgery), including mortality, thromboembolic events (transient ischaemic attack [TIA], stroke, or peripheral arterial embolism), intraoperative conversion to valve-replacement surgery, early reinterventions, and early recurrent aortic regurgitation (AR) grade 2 or higher, and (ii) AV reintervention-free (AVRF) survival, defined as survival without having undergone reintervention at follow-up. Reintervention was defined as any repeat surgical or transcatheter procedure on the AV or root, including valve replacement, redo VSARR, or full root replacement. Conversion was defined as valve replacement performed after completion of VSARR, excluding cases where VSARR was abandoned after primary intraoperative valve assessment. A composite end-point of early adverse events was created and included all-cause mortality, reintervention, thromboembolic events, intraoperative conversions to valve replacement, and recurrent AR grade 2 or higher, as these outcomes collectively reflect early procedural safety and surgical expertise. Secondary outcomes comprised the separate end-points of the early composite end-point, long-term survival, long-term freedom from reintervention, and long-term AVRF.

### Statistical analysis

Baseline characteristics were presented as means and standard deviations (SDs) or medians and interquartile ranges (IQRs). Distribution was tested using the Kolmogorov-Smirnov test. Annual institutional case volume was calculated as the mean number of VSARR procedures per year of registry participation per centre. For descriptive purposes, the baseline and procedural characteristics, as well as early and late outcomes, were presented for the overall sample and retrospectively stratified by the AVRF-derived threshold (see Results section). Comparisons between categorical data were made using the χ^2^-test, while continuous data were compared using Mann-Whitney U or Student’s T-test for continuous data, depending on their distribution.

Follow-up completeness was calculated using the Modified Clark’s Completeness Index C*.^11^^,^^12^

Long-term outcomes were analysed using time-to-event methods. Overall survival rates were assessed using Kaplan-Meier estimates and compared using Cox proportional hazards models with the frailty parameter (centre) as a random-effects term.^13^ The proportional hazards assumption for EuroSCORE II was evaluated using Schoenfeld residuals. Cumulative incidence functions (CIFs) were plotted to present absolute risks of reintervention and death.

The method to derive the threshold that could define a high-volume centre was previously described in detail and validated by multiple studies.^6^ In short, for early outcomes, the rate of the primary end-point was plotted on the y-axis and the annual institutional case load was plotted on the x-axis, after which a restricted cubic spline (RCS) model was applied, consisting of 3 knots (for model optimization). Then, for long-term outcomes, a hazard ratio (HR) was calculated per centre, using the adjusted survival of the overall sample as reference, excluding the centre of interest, per calculation (for one specific centre). The HR per centre is then plotted on the y-axis, with the centre’s corresponding annual case volume on the x-axis. Uncertainty margins (95% CIs) were recalculated to “variance” (squared SD). In the figures, smaller spheres indicate larger variance (and more uncertainty) and therefore less weight in the analyses. The potential non-linear association between annual case volume and the HR per centre was subsequently investigated using an RCS model. For model optimization, these analyses were performed for centres that contributed more than 30 cases per centre in total. Additionally, as a sensitivity analysis, the data of centres contributing 0-10, 10-20, and 20-30 cases to the registry were merged as if being one centre and subsequently entered into the model.

In line with previous studies aimed at the determination of a case volume that can define a high-volume centre,^5–9^ the threshold was determined by the “elbow of the curve.”^14^^,^^15^ At this point on the curve, an increase in case volume no longer yields a significant improvement in the survival rate.^16^

For the current analyses, we used R Statistics Version 3.6.0 (R foundation, Vienna, Austria), using the “survival,” “ggplot2,” “pathviewr” (for the elbow analysis), and “discfrail” (frailty Cox model) packages.

### Missing data

Variables with missing data are indicated in the tables. Across all baseline characteristics used in the outcome analyses, missing data resulted in the exclusion of fewer than 5% of patients; therefore, complete-case analysis was performed. For survival analyses, patients with missing follow-up data for the end-point of interest were censored at their last known follow-up date.

## Results

### AVRF survival

**Figure 1.**
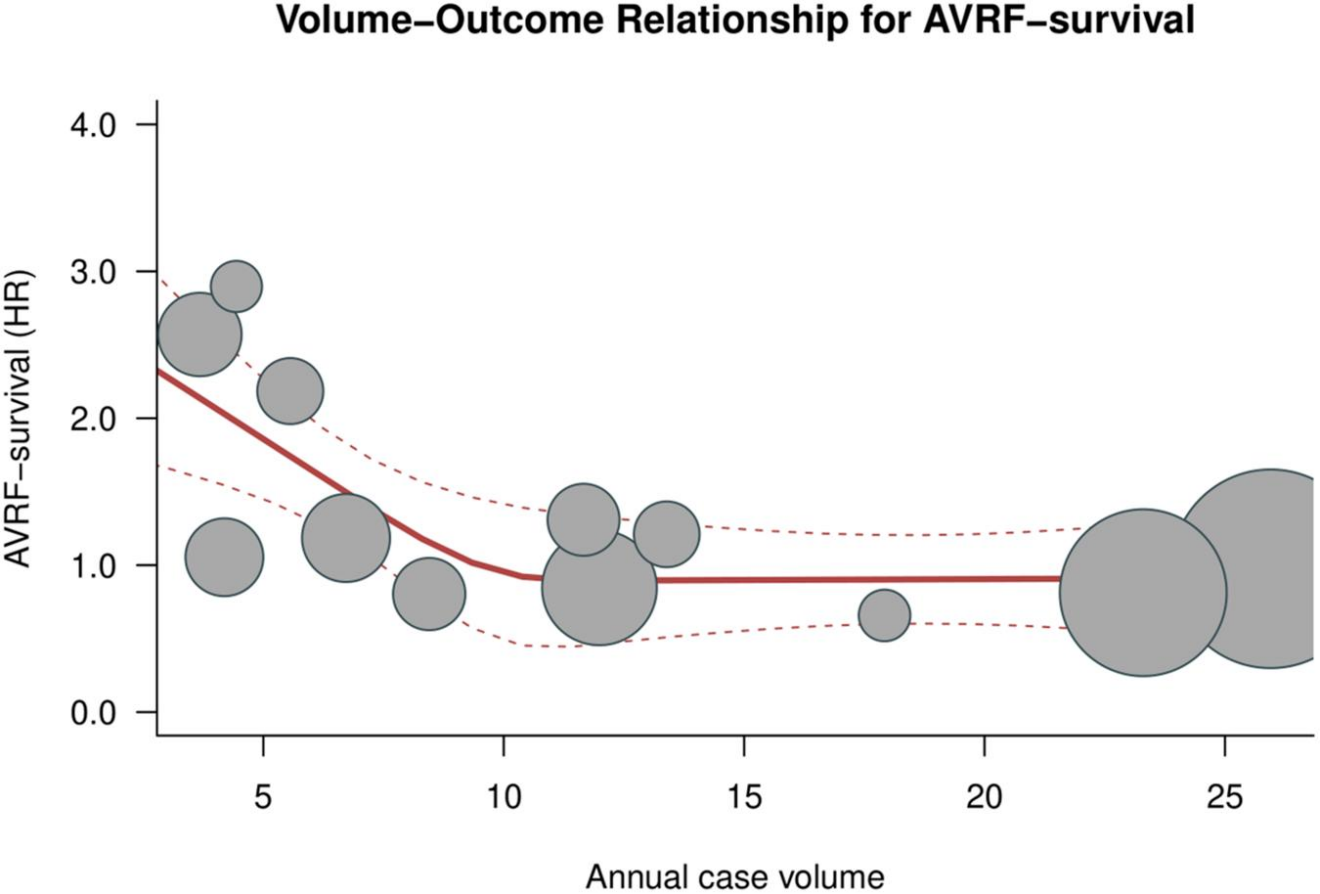
Volume-outcome Association for Long-term Aortic Valve Reintervention-free Survival, EuroSCORE II-adjusted (*n* = 12; 2226 Patients; *P* = .0023). Abbreviations: AVRF, aortic valve reintervention-free; HR, hazard ratio.

The primary long-term outcome of the study was the AVRF-survival. Cox proportional hazards analyses demonstrated a significant non-linear association between annual case-volume and AVRF-survival in centres that performed >30 cases in the registry (*P* = .0023; **Figure 1**), with an elbow at 11 cases/year (95% CI, 10-12). Based on this analysis, the threshold defining a high-volume centre was determined and the cohort was stratified into patients undergoing VSARR in centres performing <12 or ≥12 cases/year. Their baseline characteristics can be appreciated in **Tables 1 and 2** in retrospect.

**Table 1.** Baseline Patient Characteristics

Variable	Overall (*N* = 2668)	<12 p/y (*n* = 1348)	≥12 p/y (*n* = 1320)	*P*-value^a^
Demographics				
Age at surgery, years, mean (SD)	51.0 (SD: 14.2)	52.0 (SD: 13.8)	50.0 (SD: 14.6)	<.001
Male sex, *n* (%)	2285 (85.6)	1134 (84.1)	1151 (87.2)	.024
Female sex, *n* (%)	383 (14.4)	214 (15.9)	169 (12.8)	
EuroSCORE II, mean (SD)	2.62 (SD: 2.03)	2.60 (SD: 2.06)	2.64 (SD: 2.01)	.646
Preoperative comorbidities, *n* (%)				
Hypertension^b^	788 (45.8)	377 (52.8)	411 (40.8)	<.001
Pulmonary hypertension^b^				
No (PAP ≤ 30 mmHg)	2450 (94.1)	1238 (93.4)	1212 (94.8)	.223
Moderate (31-55 mmHg)	134 (5.1)	78 (5.9)	56 (4.4)	
Severe (> 55 mmHg)	20 (0.8)	10 (0.8)	10 (0.8)	
COPD^b^	102 (3.8)	66 (4.9)	36 (2.7)	.003
Critical preoperative state^b^	5 (0.2)	5 (0.4)	0 (0)	.062
Insulin-dependent diabetes^b^	36 (1.4)	30 (2.2)	6 (0.5)	<.001
Non-insulin dependent diabetes^b^	52 (3.1)	19 (2.7)	33 (3.4)	.370
Dialysis^b^	4 (0.2)	2 (0.1)	2 (0.2)	1.000
Hereditary thoracic aortic disease^b^	396 (14.8)	177 (15.3)	189 (15.3)	.982
Extracardiac arteriopathy^b^	66 (2.5)	44 (3.3)	22 (1.7)	.008
Cardiac history, *n* (%)				
Previous cardiac surgery^b^	175 (6.6)	58 (4.3)	117 (8.9)	<.001
Prior myocardial infarction^b^	27 (1)	20 (1.6)	5 (0.4)	.001
Endocarditis^b^	13 (0.5)	4 (0.3)	9 (0.7)	.156
Cardiac function, *n* (%)				
LVEF^b^				
Good (> 50%)	2263 (86.7)	1120 (85.5)	1143 (88)	.235
Moderate (31%-50%)	322 (12.3)	175 (13.4)	147 (11.3)	
Poor (21%-30%)	22 (0.8)	14 (1.1)	8 (0.6)	
Very poor (≤ 20%)	2 (0.1)	1 (0.1)	1 (0.1)	
NYHA class^b^				
I	1448 (55.9)	657 (50.5)	791 (61.3)	<.001
II	915 (35.3)	505 (38.8)	410 (31.8)	
III	214 (8.3)	129 (9.9)	85 (6.6)	
IV	13 (0.5)	9 (0.7)	4 (0.3)	

Missing values: hypertension (*n* = 946), pulmonary hypertension (*n* = 64), COPD (*n* = 6), critical state (*n* = 9), insulin-dependent diabetes (*n* = 6), non-insulin dependent diabetes (*n* = 994), dialysis (*n* = 5), extracardiac arteriopathy (*n* = 7), previous cardiac surgery (*n* = 16), prior myocardial infarction (*n* = 11), endocarditis (*n* = 8), LVEF (*n* = 59), and NYHA class (*n* = 78).

a Calculated for <12 versus ≥12 cases/year. Data are presented as mean (SD) or number (percentage).

b Percentages calculated based on available data for each variable, excluding missing values.

Abbreviations: LVEF, left ventricular ejection fraction; NYHA, New York Heart Association, PAP, pulmonary artery pressure.

**Table 2.** Procedural Characteristics

Variable	Overall (*N* = 2668)	<12 p/y (*n* = 1348)	≥12 p/y (*n* = 1320)	*P*-value^a^
Operative characteristics, *n* (%)				
Elective procedure	2668 (100)			
Aortic valve leaflet morphology				.182
Tricuspid	1664 (62.3)	864 (64.1)	800 (60.6)	
Bicuspid	966 (36.2)	468 (34.7)	498 (37.7)	
Unicuspid	30 (1.1)	11 (0.8)	19 (1.4)	
Quadricuspid	4 (0.2)	3 (0.2)	1 (<0.1)	
CPB time (mean (SD), min)	168.1 (SD: 44.6)	168.6 (SD: 48.2)	167.7 (SD: 41.6)	.367
*Total*	*1281*	*567*	*714*	
ACC time (mean (SD), min)	138.5 (SD: 36.4)	142.8 (SD: 41.3)	134.3 (SD: 30.3)	<.001
*Total*	*2612*	*1297*	*1315*	
Additional cross-clamping session	204 (7.7)	115 (8.6)	89 (6.7)	.081
*Total*	*2664*	*1344*	*1320*	
Concomitant procedures, *n* (%)				
CABG	190 (7.1)	108 (8.0)	82 (6.2)	.071
Aortic (hemi-)arch	138 (5.2)	98 (7.3)	40 (3.0)	<.001
Mitral valve surgery	137 (5.1)	60 (4.5)	77 (5.8)	.106
MAZE	107 (4.0)	57 (4.2)	50 (3.8)	.562
Total concomitant procedures	675	348	327	.506
Reopening for bleeding, *n* (%)^b^	229 (8.6)	126 (9.3)	103 (7.8)	.155
Intraoperative conversion to valve replacement, *n* (%)^b^	13 (0.5)	9 (0.7)	4 (0.3)	.174

Missing values: aortic valve leaflet morphology (*n* = 4), CPB time (*n* = 1387), ACC time (*n* = 56), additional cross-clamping session (*n* = 4), and concomitant procedures (*n* = 4).

a Calculated for <12 versus ≥12 cases/year. Data are presented as mean (SD) or number (percentage).

b Percentages calculated based on available data for each variable, excluding missing values.

*Total:* total number of patients included in the analysis.

Abbreviations: ACC, aortic cross-clamping; CABG, coronary artery bypass grafting, CPB, cardiopulmonary bypass.

### Patient and procedural characteristics

Baseline patient characteristics are presented in **Table 1**. Mean age was 51.0 (SD: 14.2) years; 85.6% were male. The LVEF prior to surgery was good (>50%) in 86.7% of patients. **Table 2** shows the procedural characteristics. Valve morphology was predominantly tricuspid (62.3%), followed by bicuspid (36.2%). Only aortic cross-clamping time and concomitant aortic (hemi-)arch surgery showed a statistically significant difference.

### Early outcomes and V-O association

Due to low early adverse event rates, only observational analyses were made. Early mortality occurred in 0.86% (23/2668) of patients, and early AV-related reintervention in 0.49% (13/2665). Thromboembolic events were reported in 1.64% of all patients (43/2663; stroke *n* = 25; TIA *n* = 12; peripheral embolism *n* = 6). Thirteen intraoperative conversions to valve replacement were performed; 9 in the <12 cases/year group and 4 in the ≥12 cases/year group (**Table 2**; *P* = .174).

**Figure 2.**
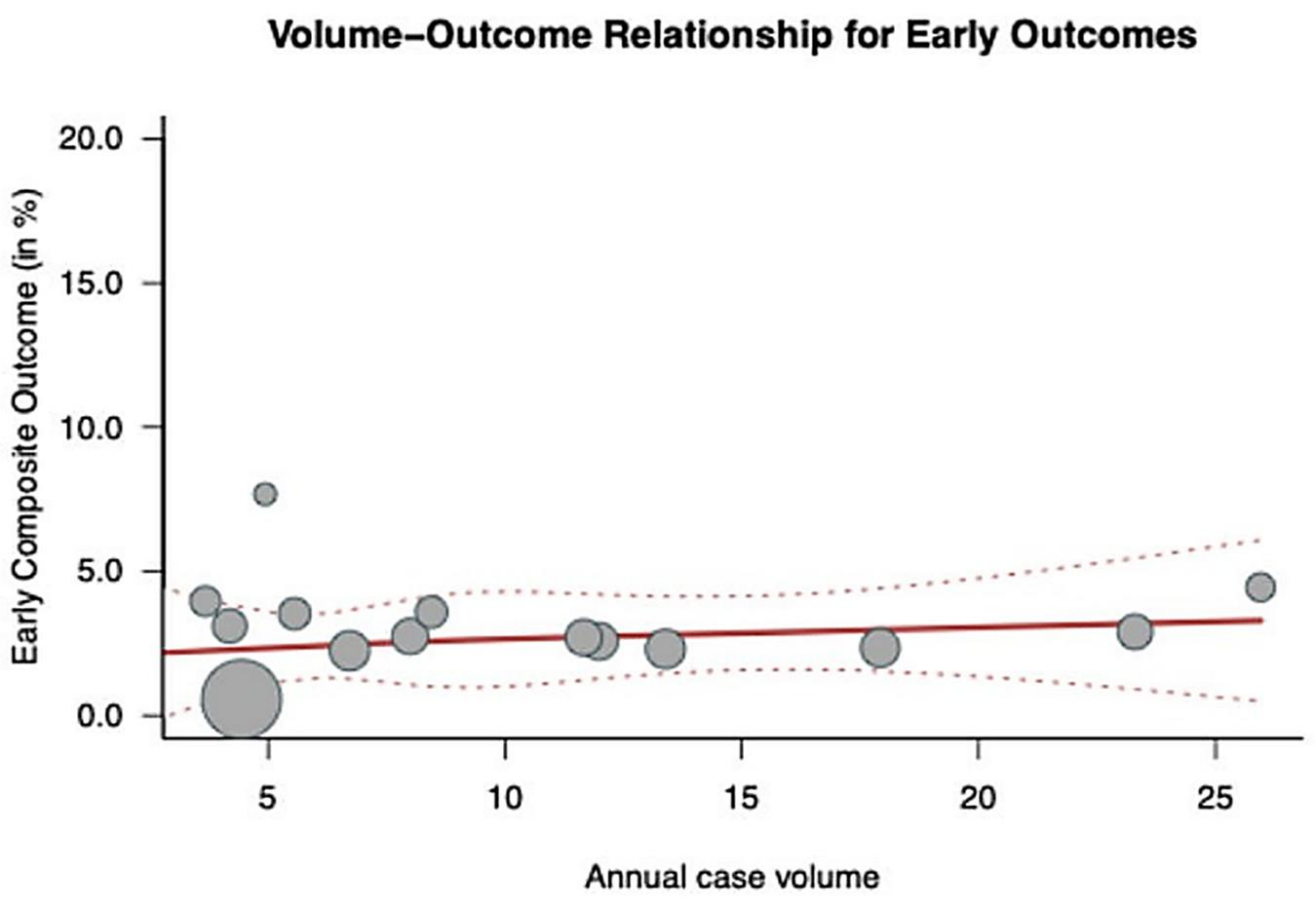
Volume-outcome Association for the Primary Early Composite End-point, EuroSCORE II-adjusted (*n* = 14; 2352 Patients; *P* = .8003).

Early recurrent AR grade ≥2 occurred in 2.3% (59/2541) of patients (grade 2: *n* = 57; grade 3: *n* = 2) and was significantly more frequent in centres performing <12 cases/year (48/1239 vs 11/1302; *P* < .001). Post-hoc analyses similarly showed higher early AV-related reintervention risks in lower-volume centres, 0.82% (11/1337; <12 cases/year) versus 0.15% (2/1318; ≥12 cases/year) (*P* = .014). Early mortality (1.0%; 14/1344 versus 0.69%; 9/1311; *P* = .319) and thromboembolic events (1.49%; 20/1344 versus 1.59%; 21/1319; *P* = .875) did not differ significantly between lower- and higher-volume centres. No significant non-linear association between annual case volume and the early composite outcome was observed in the analysis of centres contributing >30 cases (*P* = .8003; **Figure 2**).

### Long-term survival and freedom from reintervention

**Figure 3.**
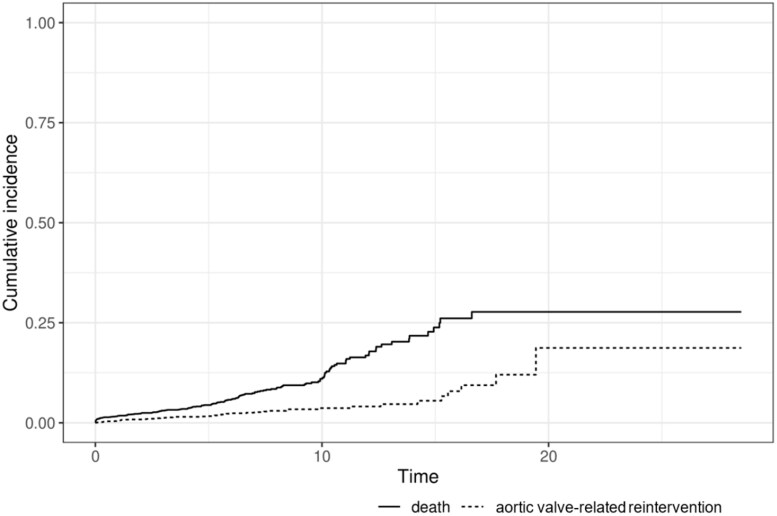
Cumulative Incidence Functions of Death and Aortic Valve-related Reintervention for the Overall Cohort.

Overall median follow-up was 3.7 years (IQR 0.9-6.7 years) with a follow-up duration of 11 960.68 patient years. Median follow-up was 2.47 years (IQR: 0.21-5.37) in <12 cases/year centres and 5.03 (IQR 1.82-8.23) in ≥12 cases/year centres. Follow-up completeness was 55.61% for the entire cohort. Kaplan-Meier analyses show the long-term survival across the cohort in **Supplementary Material S2**; the estimated overall survival of the entire cohort was 98.4% (95% CI, 97.8-99.0%), 95.8% (95% CI, 94.8-96.8%), and 87.1% (95% CI, 84.6-89.6%) at 1, 5, and 10 years, respectively. **Figure 3** shows the CIFs of death and AV-reintervention. Linearized incidence rates of mortality and AV-related reoperation are reported in **Table 3**.

**Table 3.** Overall and Stratified Linearized Incidence Rates of Mortality and AV-related Reoperation

Outcome	Overall (*N* = 2668)	<12 p/y (*n* = 1348)	≥12 p/y (*n* = 1320)
Mortality (events/total PY)	164/11 960.88	53/4570.82	111/7389.86
Mortality rate (/100 PY)	1.37	1.15	1.50
Reoperation (events/total PY)	144/11 960.88	82/4570.82	62/7389.86
Reoperation rate (/100 PY)	1.20	1.79	0.83

Abbreviation: PY, person years.

### Sensitivity analyses

Sensitivity analyses, including centres contributing fewer than 30 cases in total to the registry, demonstrated consistent results (**Supplementary Materials S3 and S4**), and the identified volume thresholds were robust across various modelling strategies. The predictive performance of EuroSCORE II was evaluated for survival using Cox regression. EuroSCORE II was significantly associated with mortality (HR 1.19, 95% CI, 1.15-1.24, *P* < .001) and AVRF (HR 1.14, 95% CI, 1.09-1.18, *P* < .001), not with reintervention (HR 1.03, 95% CI, 0.95-1.11, *P* = .520). Additionally, the proportional hazards assumption for EuroSCORE II was not violated for both mortality (*P* = .138) and reintervention (*P* = .150). This supports the validity of the time-constant HRs applied in the primary Cox regression models.

## Discussion

Valve-sparing aortic root replacement is a safe and effective procedure in contemporary practice, with low early complication rates and durable long-term outcomes.^17–21^ This is particularly relevant given the relatively young age of our patient population and the long life expectancy after a successful procedure. The 2025 ESC/EACTS Guidelines for Valvular Heart Disease recognize the growing role of VSARR in appropriately selected patients.^22^ This procedure is increasingly performed in dedicated centres, reflecting a possible shift from replacement to repair in aortic root surgery^17–21^

Our analysis showed a significant non-linear V-O association for long-term AVRF-survival within the HVS AV Database, in which institutional annual volumes ranged from 3 to 25 procedures per year. While early outcomes were uniformly low, centres performing ≥12 VSARR procedures annually were associated with superior long-term outcomes. This suggests experience and institutional exposure contribute to success but should be interpreted in context. Our findings on exposure are consistent with earlier analyses in other surgical procedures where similar volume thresholds have been identified.^5–9^

The registry reflects a historical cohort spanning over 25 years, during which annual VSARR volumes increased, particularly in the last decade. As a result, the association observed here is shaped by evolving surgical practice, patient selection, and perioperative management across time. Moreover, the registry includes exclusively David and Yacoub procedures; other valve-sparing strategies, such as isolated AV repair (AVP) or more complex AVP techniques involving extensive leaflet repair or double annuloplasty, were not included. Proficiency in David or Yacoub techniques does not automatically translate to expertise in AVP, and vice versa, and outcomes observed in our study cannot be generalized across all forms of valve-sparing surgery.

Our findings must be viewed considering several important nuances. First, referral and acceptance practices may differ between centres. Highly experienced, high-volume institutions may treat patients with more complex pathology or borderline indications and may still achieve durable outcomes despite higher inherent procedural difficulty. Conversely, some centres may concentrate on less complex cases. These centre-specific patterns cannot currently be quantified in the registry, therefore the association should not be interpreted as a sole causal effect of volume.

Current guidelines recommend that complex procedures such as VSARR be performed in Heart Valve Centres with high procedural volumes and excellent clinical outcomes.^22^

Although no specific threshold is provided, they advocate for a network model in which complex interventions are performed in the upper quartile of centres by volume and performance. Our results suggest, within the limits of this dataset, that an annual case volume of ≥12 may be beneficial to valve-durability and patient longevity. This number should not be interpreted as a rigid cutoff, but rather as an opportunity for lower-volume centres to reflect on their current practice and consider strategies for structured growth. Centres aiming to build expertise in VSARR should first gain experience in AV and root surgery. They can then consider starting with less complex, elective VSARR cases, such as isolated aneurysms without significant valve pathology, and gradually increasing procedural exposure and complexity. Ensuring consistent exposure to the procedure is key to retaining improved patient outcomes.

The trend towards valve preservation is reinforced by our findings. Valve-sparing aortic root replacement avoids prosthetic valve-related complications, maintains native valve haemodynamics, and offers excellent short- and long-term outcomes, especially in high-volume centres. VSARR offers a compelling alternative to valve replacement, particularly in younger patients where prosthetic valve durability and anticoagulation may be of concern.

### Limitations

Several limitations of this study should be acknowledged. First, as a retrospective registry analysis, selection bias and residual confounding remain possible, despite the adjustment for EuroSCORE II.

Second, the registry spans over 25 years during which changes in techniques, patient selection, and perioperative care may influence comparability across centres. In addition, annual institutional volume was calculated as a mean across each centre’s period of participation in the registry. This static measure does not capture temporal changes in expertise or innovations in surgical and peri-/postoperative care, potentially biasing results towards centres with long-standing programs. Third, procedural complexity (eg, severity and morphology of aortic insufficiency or the number of leaflets treated) was not accounted for in our analysis. Although these factors may influence surgical difficulty and long-term outcomes, no standardized or validated method exists to reliably quantify procedural complexity. This limits our ability to determine whether the V-O association reflects true effects of procedural exposure or differences in patient populations. Fourth, surgeon-specific volumes were unavailable due to privacy restrictions, preventing assessment of the relative contributions of operator- versus institutional-level experience. Finally, modelling required the aggregation of low-contributing centres into surrogate categories. Although sensitivity analysis supported the findings, this may obscure heterogeneity between individual centres.

## Conclusion

In this multicentre registry-based analysis, a significant association between institutional case volume and long-term AVRF survival following elective VSARR was observed. Based on our analyses, an institutional volume of 12 or more elective VSARRs annually is associated with long-term valve durability and survival. This high-volume threshold represents a benchmark for centres and surgeons already experienced in AV and/or root surgery, not a minimum for initiating VSARR, and should be interpreted within the registry’s limits. Further research incorporating procedural complexity, surgeon-level data, and contemporary populations is required to refine these benchmarks.

## Supplementary Material

ezag177_Supplementary_Data

## Data Availability

Data underlying this article were provided by the Heart Valve Society by permission. Data will be shared on request to the corresponding author with permission of the Heart Valve Society.

## Abbreviations

AV: aortic valve
HR: hazard ratio
HVS: Heart Valve Society
IQR: interquartile range
RCS: restricted cubic splines
SD: standard deviation
VSARR: valve-sparing aortic root replacement
V-O: volume-outcome
