# Supplementary material for: Valve-sparing Aortic Root Replacement: Defining High-volume Centres Using Prospective Data
Source: Eur J Cardiothorac Surg. 2026 May 22;68(6):ezag177. doi: 10.1093/ejcts/ezag177 (PMC13282078; doi:10.1093/ejcts/ezag177)
Supplement: ezag177_Supplementary_Data [file ezag177_supplementary_data.pdf]

SUPPLEMENTARY MATERIAL

Supplementary Material S1. CONSORT flow diagram on patient selection from the Heart Valve Society Aortic Valve Database.

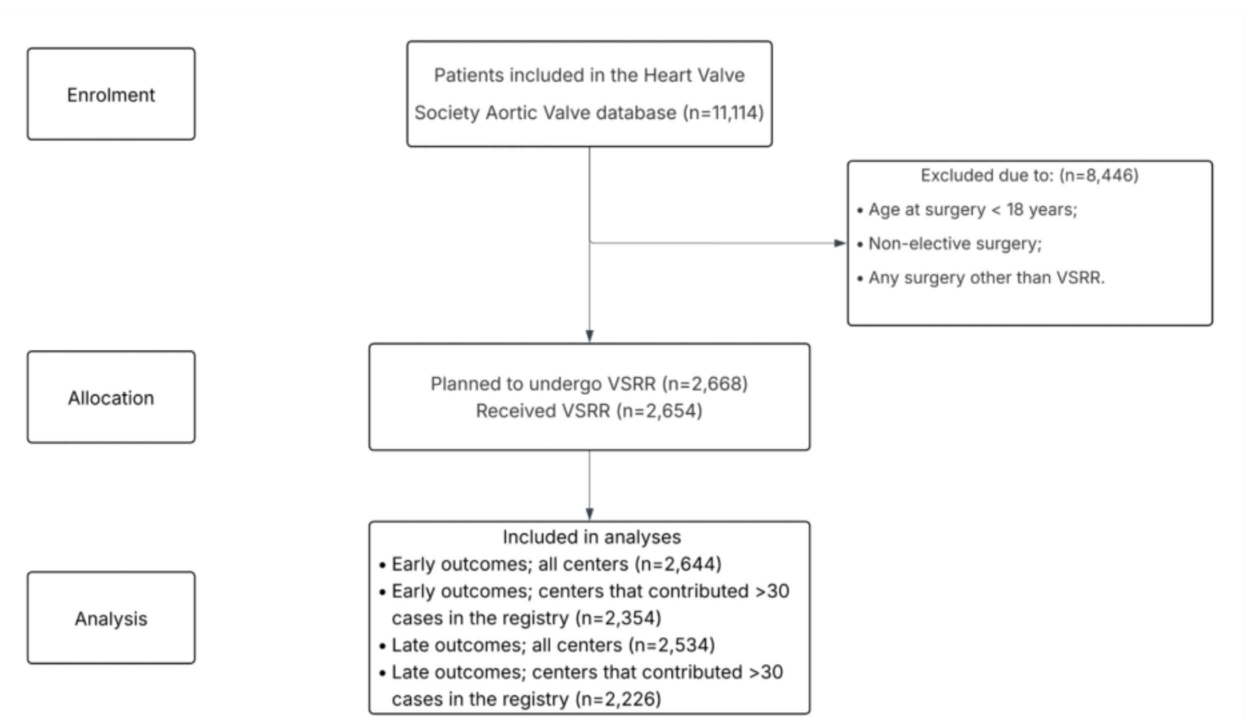

Supplementary Material S2. Kaplan-Meier estimate for overall survival.

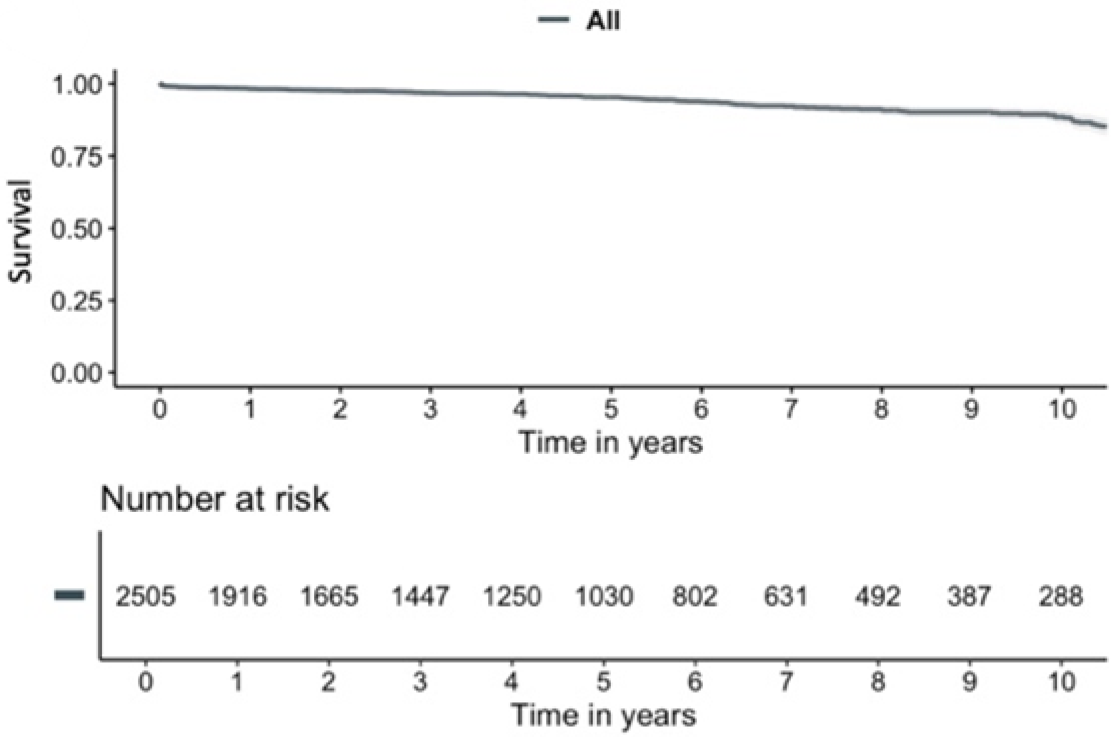

Supplementary Material S3. Volume-outcome association for AVRF-survival in restricted cubic spline analysis, incorporating centres performing 0-10, 10-20, and 20-30 cases per year and incorporated into the model as single surrogate centres (resulting in three additional circles in the graph) (p<0.0001).

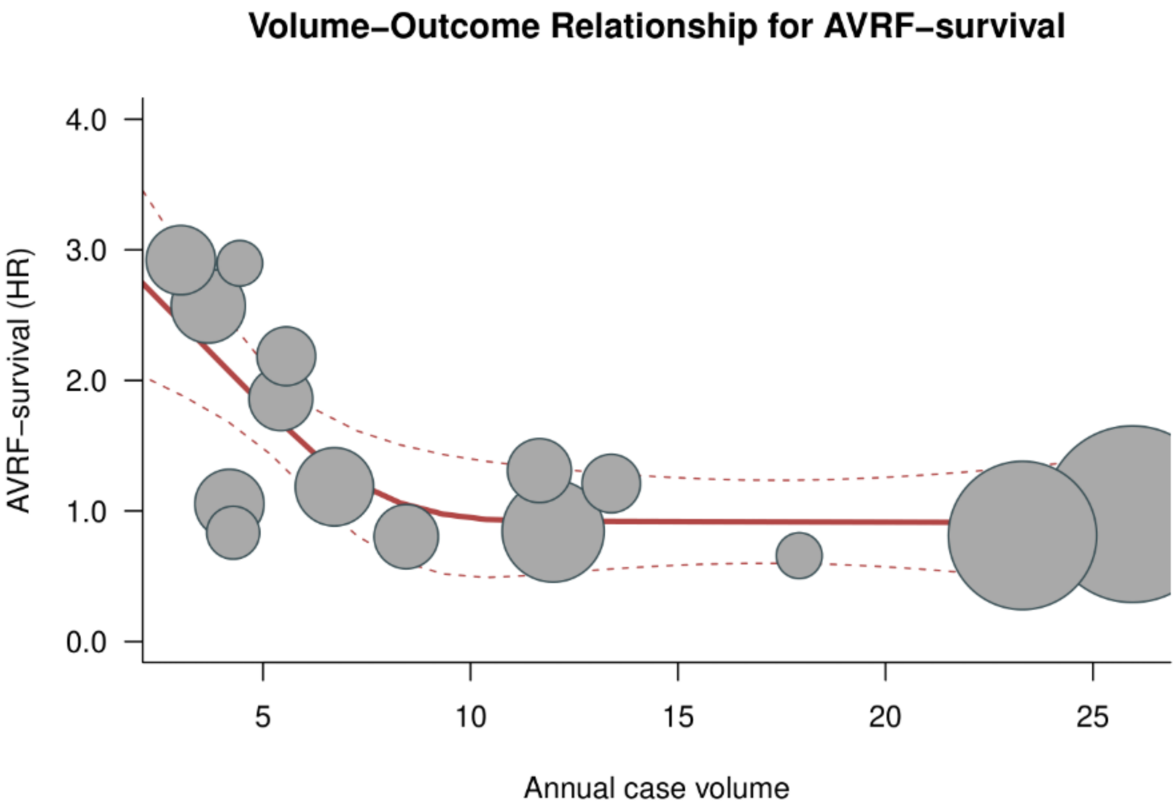

Supplementary material S4. Volume-outcome association for early outcomes; pooled results of all centres (p=0.7110).

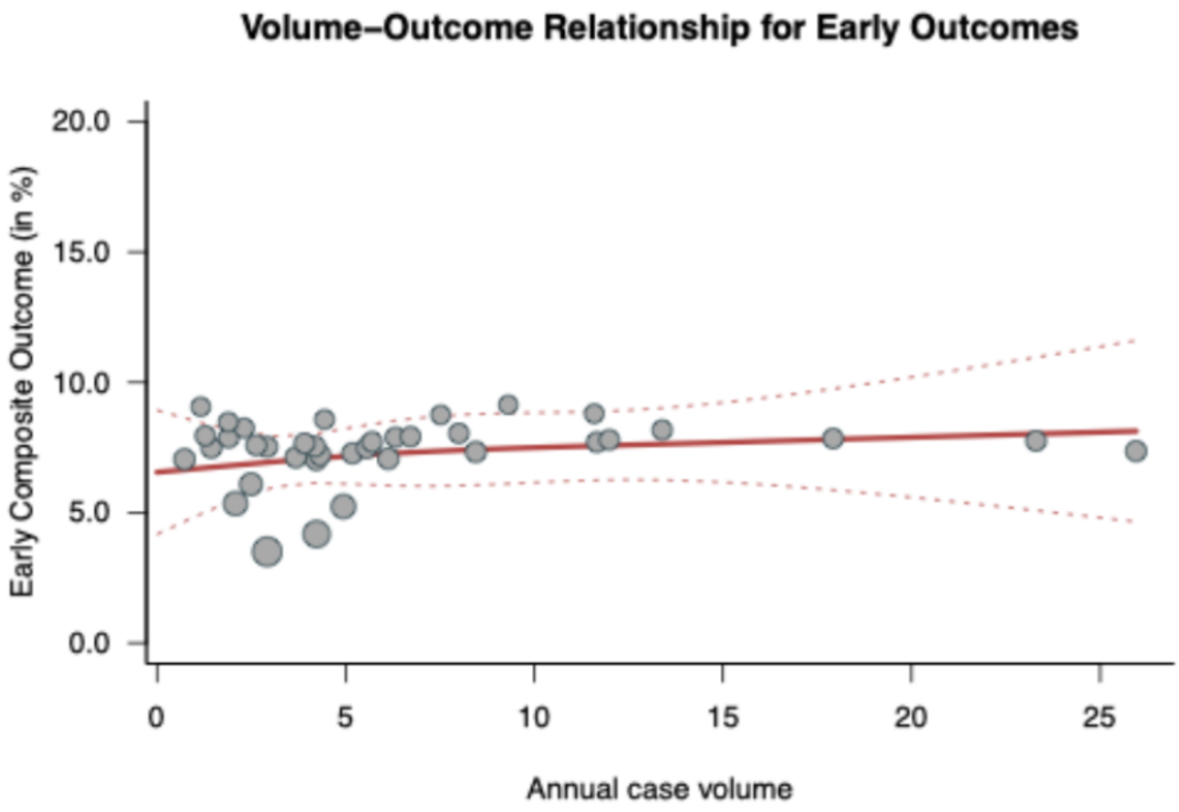

Supplementary Material S5. Full list of the Aortic Valve Research Network Investigators.

Collaborators: Claudia Romagnoni, Frederiek de Heer, Jesper Hjortnaes, Adrián Kolesár, Alejandro Crespo de Hubsch, Christian Dinges, Jaroslav Hlubocky, Carlotta Brega, Ruggero de Paulis, Mauro Masat, Francesco Patane, Matteo Pettinari, Maciej Matuszewski, Gianclaudio Mecozzi, Jan Nijs, Marek Jasinski, Javier Estigarribia, Massimo Mariani, Pallav Shah, Diana Aicher, Didier Chatel, Aayush Poddar, Vladislav Aminov, Eric Bergoend, Aude Boignard, Guillaume Geuzebroek, Takeshi Miyairi, German Chaud, Ignacio Bibiloni, Evaldas Girdauskas, Guido van Aarnhem, Said Soliman, Mladen Kocica, Alberto Forteza, Carlos Porras, Plamen Panayotov, Yutaka Okita, Thierry Bourguignon, Corinne Coulon, Nadia Mansour, Kathy Louro, Vincent Chauvette, Bart Meuris, Davor Baric, Daniel Unic, Olivier Bouchot, Mikita Karalko, Pavel Žáček, Rubina Rosa, Andrea Mangini, Tomáš Toporcer, Johannes Steindl, Robert Novotny, Andrey Slautin, Giulio Folino, Fabrizio Ceresa, Carlijn van der Ven, Patrick Yiu, Ryan Accord, Cristobal Alvarado, Joaquin Gundelach, Gregorio Rábago, Leona Schultz, Dejan Lazovic, Carlos Martin, Gemma Sánchez-Espín, Milen Slavov
